# Clinical outcomes of patients with giant cell arteritis treated with tocilizumab in real-world clinical practice: decreased incidence of new visual manifestations

**DOI:** 10.1186/s13075-020-02377-8

**Published:** 2021-01-06

**Authors:** Sebastian Unizony, Timothy J. McCulley, Robert Spiera, Jinglan Pei, Paris N. Sidiropoulos, Jennie H. Best, Christine Birchwood, Andrey Pavlov, John H. Stone

**Affiliations:** 1 Massachusetts General Hospital Rheumatology Unit, Harvard Medical School, 55 Fruit Street, Boston, MA 02114-2696 USA; 2 Wilmer Eye Institute, Johns Hopkins University School of Medicine, Baltimore, MD USA; 3 Department of Medicine, Hospital for Special Surgery, New York, NY USA; 4 Genentech, Inc., South San Francisco, CA USA; 5 Everest Clinical Research, Markham, ON Canada

**Keywords:** Giant cell arteritis, Tocilizumab, Real-world study, Visual manifestations, Polymyalgia rheumatica

## Abstract

**Background:**

Placebo-controlled clinical trials have demonstrated the efficacy of tocilizumab (TCZ) for remission maintenance and glucocorticoid sparing in patients with giant cell arteritis (GCA). However, limited data exist on the effectiveness and safety of TCZ for GCA in real-world clinical practice.

**Methods:**

This was a retrospective, single-center analysis of patients with GCA treated with intravenous or subcutaneous TCZ (2010–2018). Outcomes evaluated before and after TCZ initiation included occurrence of flare, time to flare, annualized flare rate, flare characteristics (i.e., polymyalgia rheumatica [PMR] symptoms, cranial manifestations), prednisone use, and safety. Flare was defined as the recurrence of unequivocal GCA manifestations requiring treatment intensification. Subgroup analyses of patients with PMR or visual manifestations at GCA diagnosis were performed.

**Results:**

Sixty patients with GCA were included. The median (IQR) disease duration before and after the start of TCZ was 0.6 (0.2–1.6) and 0.5 (0.3–1.4) years, respectively. At least 1 flare was observed in 43 patients (71.7%) before and in 18 (30.0%) after TCZ initiation. Median (IQR) time to flare was 0.5 (0.3–0.7) years before TCZ treatment and 2.1 (0.6–2.6) years after TCZ initiation (HR 0.22; 95% CI 0.10–0.50; *p* = 0.0003). The annualized flare rate significantly decreased following TCZ use (before TCZ 1.4 [95% CI 1.0–2.1]; after TCZ 0.6 [95% CI 0.3–1.0] events/year; *p* < 0.001). Similar improvements were observed in patients with visual manifestations or PMR symptoms at GCA diagnosis. TCZ reduced the incidence of new visual manifestations, and no flares associated with permanent vision loss occurred while patients were receiving TCZ. Mean (SD) prednisone dose at TCZ onset and at the end of follow-up was 30 (18.3) and 5 (6.9) mg/day, respectively (*p* < 0.0001). After TCZ initiation, 46.6% of patients successfully discontinued prednisone. The incidence of adverse events, primarily attributed to glucocorticoids, was similar before and after TCZ initiation.

**Conclusions:**

In this real-world setting, TCZ improved GCA clinical outcomes significantly and demonstrated effectiveness in the subgroups of patients with PMR symptoms and GCA-related visual manifestations at GCA diagnosis. No new cases of blindness occurred after TCZ initiation. Adverse events, many attributable to glucocorticoids, were comparable before and after TCZ treatment.

## Background

Until recently, treatment for patients with giant cell arteritis (GCA) entailed prolonged courses of glucocorticoids (e.g., prednisone), which are adequate for inducing disease remission but are not effective in preventing disease relapse in the majority of patients [1–5]. In addition, prolonged tapering of glucocorticoids typically exposes patients to high cumulative glucocorticoid doses, frequently leading to treatment-related toxicities such as infection, osteoporosis, cataracts, diabetes, and hypertension [6–8]. A new treatment option is available after randomized clinical trials demonstrated that interleukin 6 (IL-6) receptor blockade with tocilizumab (TCZ) in combination with shorter glucocorticoid courses is efficacious for maintaining disease remission and sparing the use of glucocorticoids in patients with new-onset or relapsing GCA [5, 9].

Real-world data on the effectiveness and safety of TCZ in GCA are currently limited [10–12]. Although the clinical course with regard to the timing and characteristics of disease flares in patients with GCA treated with glucocorticoids alone has been studied in detail [2–4], longitudinal analyses of patients with GCA receiving TCZ in a real-world setting have not been performed. Furthermore, some evidence suggests that TCZ is effective in controlling polymyalgia rheumatica (PMR), a comorbid condition observed in nearly half of patients with GCA [13, 14]. It is unknown, however, whether GCA patients with PMR symptoms at diagnosis respond differently to TCZ compared with those without such symptoms and whether IL-6 blockade reduces the incidence of PMR during subsequent disease relapses. Finally, anterior ischemic optic neuropathy (AION) has been reported in a patient receiving TCZ [5], but the incidence of GCA-related visual manifestations during disease flare in patients receiving IL-6 blockade therapy is unknown.

The primary objective of the present study was to evaluate the longitudinal effectiveness and safety of TCZ in patients with GCA in real-world clinical practice. In addition, we aimed to determine whether the presence of PMR and GCA-related visual symptoms at baseline influences patient response to treatment. Finally, we assessed the clinical characteristics of disease flares occurring despite TCZ therapy, with a particular focus on visual manifestations.

## Methods

### Study design and patient population

A retrospective analysis was conducted using electronic medical records (EMRs) from the first 60 patients who had received a diagnosis of GCA and were treated with intravenous (IV) or subcutaneous (SC) TCZ by rheumatologists at Massachusetts General Hospital (MGH;Boston, MA, USA) between 2010 and 2018. The sample size was chosen based on feasibility. All patients met the 1990 American College of Rheumatology (ACR) classification criteria for GCA. Participants in the GiACTA trial [5] were excluded from the study. Patients received TCZ at the discretion of the treating rheumatologist either because of disease relapse despite the use of other therapies (e.g., prednisone) or because they had comorbidities (e.g., poorly controlled diabetes) that required a glucocorticoid-sparing strategy.

### Study assessments

Efficacy and safety outcomes and patterns of prednisone use were evaluated before and after the initiation of TCZ by reviewing all rheumatology notes, laboratory values, and imaging studies available in each patient EMR. From disease diagnosis or referral to MGH, patients were followed at variable intervals, but mostly every 1–3 months. The primary outcome was the occurrence of disease flares, defined as the reappearance of unequivocal clinical manifestations of GCA (e.g., cranial or PMR symptoms) that required treatment intensification, regardless of the erythrocyte sedimentation rate and C-reactive protein level. Additional outcomes were time to flare, annualized flare rate, flare characteristics including PMR and visual manifestations, ability to discontinue prednisone, and occurrence of adverse events (AEs) and serious AEs (SAEs). Further subgroup analyses were performed for patients with PMR symptoms or visual manifestations at the time of GCA diagnosis. GCA-related visual manifestations assessed included diplopia, transient blurred vision, amaurosis fugax, and permanent vision loss due to AION or central retinal artery occlusion.

### Statistical analysis

Patient baseline characteristics and efficacy and safety endpoints were summarized using descriptive statistics. Hazard ratios (HRs) for time to flare were estimated from a Cox regression model with ongoing treatment (TCZ and glucocorticoid combinations), age, smoking history, and new or relapsing GCA at first MGH visit as covariates, and random patient effect. Kaplan-Meier curves were plotted for time-to-flare outcomes. Rate ratios of flares were estimated from a Poisson regression model with ongoing treatment (TCZ and GC combinations), age, smoking history, and new or relapsing GCA at first MGH visit as covariates, and random patient effect.

### Ethical considerations

The study was approved by the Partners Human Research Committee institutional review board (IRB; protocol # 2017P001636) and was conducted in accordance with the Declaration of Helsinki. All data extracted from the EMRs were stored de-identified prior to the analysis. As per our institutional IRB guidelines, this retrospective research did not require informed consent.

## Results

### Baseline demographics and clinical characteristics

Sixty patients with GCA were included in the study. Baseline patient characteristics are shown in Table 1. The majority was female (71.7%) and white (88.3%), and the mean (SD) age was 69.3 (9.4) years. Fifty (83.3%) patients were diagnosed at MGH, and 10 (16.7%) were referred to this institution after being diagnosed elsewhere and having a disease flare. TCZ treatment was introduced upfront as a glucocorticoid-sparing alternative within 10 weeks of GCA diagnosis and without preceding flare in 15 subjects (25%). The other 45 patients (75%) received TCZ either after developing 1 or more disease flares (*n* = 43) or following the occurrence of glucocorticoid-related toxicity after 10 weeks of GCA diagnosis (*n* = 2). The median (interquartile range [IQR]) disease duration before receiving TCZ was 0.6 (0.2–1.6) years. Patients received TCZ for a median (IQR) duration of 0.5 (0.3–1.4) years. Most patients received SC TCZ (*n* = 44), but 22 received IV TCZ and 6 received both SC and IV TCZ. A total of 58 patients (96.7%) received concomitant prednisone (mean [SD] dose, 30 [18.3] mg daily) at the time of TCZ initiation.

**Table 1:** Patient baseline characteristics and treatments

	All patients, *N* = 60
Age at diagnosis, mean (SD), years	69.3 (9.4)
White, *n* (%)	53 (88.3)
Female, *n* (%)	43 (71.7)
Previous or current smoking history, *n* (%)	21 (35.0)
New-onset disease, *n* (%)*	48 (80.0)
Clinical manifestations at disease onset	
Localized headache, *n* (%)	47 (78.3)
Scalp tenderness, *n* (%)	26 (43.3)
Jaw claudication, *n* (%)	31 (51.7)
PMR symptoms, *n* (%)	32 (53.3)
Visual manifestations, *n* (%)	22 (36.7)
Amaurosis fugax, *n* (%)	11 (18.3)
Transient blurry vision, *n* (%)	18 (30.0)
Diplopia, *n* (%)	2 (3.3)
Permanent vision loss, *n* (%)	8 (13.3)
AION, *n* (%)	7 (11.7)
CRAO, *n* (%)	1 (1.7)
Fever, *n* (%)	14 (23.3)
Weight loss, *n* (%)	20 (33.3)
ESR, mean (SD), mm/h	72.6 (33.6)
CRP, mean (SD), mg/L	76.1 (72.7)
Positive temporal artery biopsy, *n*/*N* (%)**	26/51 (51.0)
Large-vessel vasculitis, *n*/*N* (%)**	12/28 (42.9)
Prednisone dose at the time of GCA diagnosis, mean (SD), mg/day	54.0 (19.0)
Duration of prednisone use before TCZ initiation, median (IQR), years^†^	0.6 (0.2–1.4)
Use of other immunosuppressants before TCZ initiation, *n* (%)^‡^	14 (23.3)
Prednisone dose at TCZ initiation, mean (SD), mg/day	30.0 (18.3)
Duration of disease before TCZ initiation , median (IQR), years	0.6 (0.2–1.6)
Received TCZ upon disease onset (new-onset disease), *n* (%)^¥^	15 (25.0)
Received intravenous TCZ, *n* (%)^§^	22 (36.7)
Received subcutaneous TCZ, *n* (%)^§^	44 (73.3)
Duration of TCZ treatment, median (IQR), years	0.5 (0.3–1.4)

*AION* anterior ischemic optic neuropathy, *CRAO* central retinal artery occlusion, *CRP* C-reactive protein, *ESR* erythrocyte sedimentation rate, *GCA* giant cell arteritis, *IQR* interquartile range, *MGH* Massachusetts General Hospital, *PMR* polymyalgia rheumatica, *TCZ* tocilizumab

*At first MGH visit

**Of patients assessed. Large-vessel vasculitis was defined as the presence of circumferential wall thickening, edema, contrast enhancement, and/or ^18^fluorine-2-deoxy-d-glucose uptake in large arteries identified by cross-sectional imaging including magnetic resonance angiography, computed tomography angiography, or positron emission tomography

^†^Of 59 patients with prednisone use prior to TCZ initiation

^‡^Other immunosuppressants included methotrexate, ustekinumab, abatacept, rituximab, leflunomide, tofacitinib, and cyclophosphamide

^¥^TCZ started within 10 weeks of GCA diagnosis without preceding disease flare

^§^Six patients received both intravenous and subcutaneous TCZ

Thirty-two patients (53.3%) had PMR symptoms, and 22 patients (36.6%) had GCA-related visual manifestations at the time of GCA diagnosis. Other clinical manifestations at disease onset were headache (78.3%), jaw claudication (51.7%), scalp tenderness (43.3%), weight loss (33.3%), and fever (23.3%). Two patients presented with PMR symptoms only (no cranial symptoms). The most common visual manifestations were blurred vision (30.0%), amaurosis fugax (18.3%), and permanent vision loss from either AION or central retinal artery occlusion (13.3%).

### Incidence of flares

**Fig. 1:**
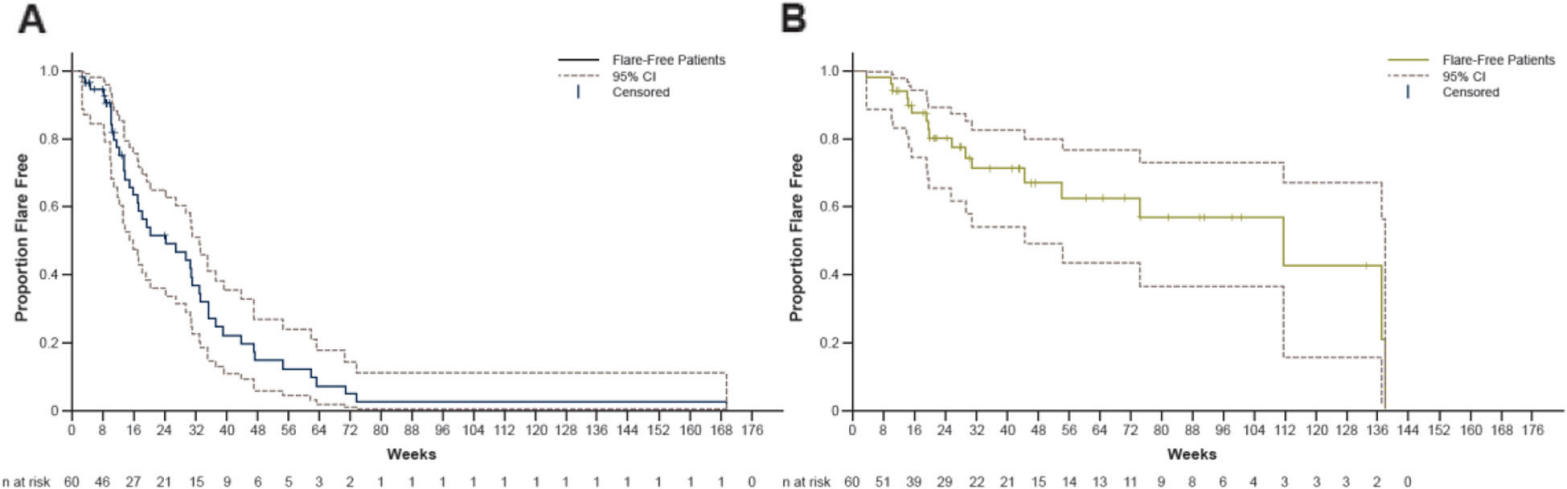
Time to first disease flare before (**a**) and after (**b**) tocilizumab initiation. Dotted lines represent 95% CIs

Overall, 43 patients (71.7%) had ≥ 1 flare before initiating TCZ. Once on TCZ therapy, ≥ 1 flare occurred in 18 patients (30.0%; Table 2). The occurrence of disease flares over time in the overall patient population is depicted in Supplemental Fig. 1. Patients had a significantly lower rate of flare after TCZ than before TCZ initiation (0.6 [95% CI 0.3–1.0] vs 1.4 [95% CI 1.0–2.1] flares per year). The annualized flare rate ratio after TCZ initiation compared with the time prior to TCZ treatment was 0.4 (95% CI 0.3–0.6; *p* < 0.001). A subgroup analysis excluding the 15 patients that received TCZ upfront showed similar results (annualized flare rate ratio 0.4 [95% CI 0.2–0.6]; *p* = 0.0002). A total of 102 flares were observed before the use of TCZ, of which 57 patients (55.9%) had PMR symptoms and 15 (14.7%) had visual manifestations. Following TCZ treatment, 37 flares occurred, of which 21 patients (56.8%) had PMR symptoms and only 3 (8.1%) had visual manifestations. Compared with the period before TCZ treatment, the time to flare was significantly longer after the patients started TCZ (HR 0.2; 95% CI 0.1–0.5; *p* < 0.001; Fig. 1).

**Table 2:** Disease flares before and after TCZ initiation

	Total patients, *N* = 60	
	Before TCZ initiation	After TCZ initiation
Follow-up time, median (IQR), years	0.6 (0.2–1.6)	0.6 (0.3–1.5)
Rate of flares per year*		
Rate (95% CI)	1.4 (1.0–2.1)	0.6 (0.3–1.0)
Rate ratio (95% CI)		0.4 (0.3–0.6)
*p* value		0.0001
Patients with ≥ 1 flare, *n* (%)	43 (71.7)	18 (30.0)
1 flare	19 (31.7)	11 (18.3)
2 flares	11 (18.3)	3 (5.0)
3 flares	4 (6.7)	3 (5.0)
≥ 4 flares	9 (15.0)	1 (1.7)
Total no. of flares	102	37
With visual manifestations, *n* (%)^†^	15 (14.7)	3 (8.1)
With PMR symptoms, *n* (%)^†^	57 (55.9)	21 (56.8)

*GCA *giant cell arteritis*, IQR* interquartile range, *PMR* polymyalgia rheumatica, *TCZ* tocilizumab

*Rates were estimated from a Poisson regression model with ongoing treatment (TCZ and prednisone combinations), age, smoking history, and new or relapsing GCA as covariates, and random patient effect

^†^Symptoms were after GCA diagnosis. Percentage of flares is out of the total number of flares

### Prednisone use

The mean (SD) prednisone dose to treat the initial presentation of the disease was 54.0 (19.0) mg/day (Table 1). Patients required prednisone for a median (IQR) period of 0.6 (0.2–1.4) years before starting TCZ. Of the 58 patients receiving prednisone at the moment of TCZ initiation (mean [SD] dose, 30.0 [18.3] mg/day), 27 (46.6%) tapered off glucocorticoids completely after beginning TCZ treatment. Before TCZ, the mean (SD) prednisone dose at which flares occurred was 10.8 (12.0) mg/day and the mean (SD) prednisone dose chosen to treat flares was 31.9 (30.0) mg/day. After TCZ initiation, the mean (SD) prednisone doses at flare and in response to flare were 11.8 (15.8) mg/day and 30.4 (25.7) mg/day, respectively. The mean (SD) prednisone dose at last follow-up was 5.0 (6.9) mg/day.

### Safety

Before TCZ initiation, 29 patients (48.3%) had 59 AEs. Fifty-two (88.1%) of these 59 AEs were attributed to glucocorticoids. The most commonly reported AEs before TCZ initiation were psychiatric complications (15.0%), impaired glucose metabolism (8.3%), and osteopenia (8.3%; Table 3). After initiating TCZ, 32 patients (53.3%) had 81 AEs, of which 36 (44.4%) were attributed to glucocorticoids, 27 (35.9%) were attributed to TCZ, and 55 (67.9%) were attributed to either glucocorticoids or TCZ. The most commonly reported AEs after TCZ initiation were cataract formation (6.7%) and pneumonia (6.7%).

**Table 3:** Adverse events

	Before TCZ initiation (*N* = 60)	After TCZ initiation (*N* = 60)
AEs reported in ≥ 2 patients, *n* (%)		
AEs attributed to glucocorticoids		
Cataract	3 (5.0)	4 (6.7)
Impaired glucose metabolism	5 (8.3)	3 (5.0)
Psychiatric complications*	9 (15.0)	1 (1.7)
Gastroesophageal reflux disease	3 (5.0)	0
Gastrointestinal hemorrhage	1 (1.7)	2 (3.3)
Myopathy	4 (6.7)	1 (1.7)
Osteopenia	5 (8.3)	1 (1.7)
Atrial fibrillation	1 (1.7)	2 (3.3)
Hypertension	1 (1.7)	2 (3.3)
Cardiac failure	2 (3.3)	0
AEs attributed to glucocorticoids and/or TCZ		
Dyslipidemia	0	2 (3.3)
Pneumonia	4 (6.7)	4 (6.7)
Urinary tract infection	1 (1.7)	2 (3.3)
Sinusitis	0	2 (3.3)
Herpes zoster infection	2 (3.3)	1 (1.7)
AEs attributed to TCZ		
Leukopenia	0	3 (5.0)
Thrombocytopenia	0	2 (3.3)
Increased transaminases	0	3 (5.0)
AEs unrelated to glucocorticoids and/or TCZ		
Osteoarthritis	0	3 (5.0)
Musculoskeletal pain	0	3 (5.0)
Deep vein thrombosis	0	2 (3.3)
Lumbar radiculopathy	0	2 (3.3)
SAEs reported in ≥ 1 patient, *n* (%)	6 (10.0)	6 (10.0)
SAEs attributed to glucocorticoids		
Diabetic ketoacidosis	0	1 (1.7)
Gastrointestinal hemorrhage	0	2 (3.3)
Osteoporotic hip fracture	1 (1.7)	0
Depression	1 (1.7)	0
Atrial fibrillation	1 (1.7)	1 (1.7)
Cardiac failure	2 (3.3)	0
SAEs attributed to glucocorticoids and/or TCZ		
*Pneumocystis jiroveci* pneumonia	1 (1.7)	0
Sepsis	0	1 (1.7)
SAEs attributed to TCZ		
Leukopenia	0	1 (1.7)
Intestinal perforation	0	1 (1.7)
SAEs unrelated to glucocorticoids and/or TCZ		
Acute kidney injury	0	1 (1.7)
Leiomyosarcoma	0	1 (1.7)
Pneumonitis	1 (1.7)	0
Pulmonary embolism	1 (1.7)	0

*AE* adverse event, *SAE* serious adverse event, *TCZ* tocilizumab

*Psychiatric complications included depression, worsening depression, insomnia, mood swings, and psychosis

Before TCZ, 6 patients (10.0%) had 8 SAEs, 7 (87.5%) of which were attributed to glucocorticoids. After initiating TCZ, 6 patients (10.0%) had 9 SAEs, of which 3 (33.3%) were attributed to TCZ, 4 (44.4%) were attributed to glucocorticoids, and 6 (66.7%) were attributed to either glucocorticoids or TCZ. Five patients (8.3%) discontinued TCZ due to an SAE (1 event each of pneumonia, sepsis, bowel perforation, severe leukopenia, and allergic reaction). Of note, 2 serious infections were reported (1 event each of *Pneumocystis jiroveci* pneumonia [before TCZ] and sepsis [after TCZ]). After the patients initiated TCZ, 1 malignancy (leiomyosarcoma) and 1 episode of diverticulitis were reported. No deaths occurred during the study period.

### Outcomes in patients with PMR symptoms at GCA diagnosis

Baseline characteristics for patients with and without PMR symptoms at GCA diagnosis are shown in Supplemental Table S1. Of the 32 patients with PMR symptoms at diagnosis, 24 (75.0%) had ≥ 1 flare before TCZ initiation and 11 (34.4%) had ≥ 1 flare after TCZ initiation (Table 4). PMR symptoms were reported in 50 of the 60 total flares (83.3%) before TCZ initiation and in 18 of the 23 total flares (78.3%) after TCZ initiation. Among the 28 patients without PMR symptoms at diagnosis, 19 (67.9%) had ≥ 1 flare before TCZ initiation and 7 (25.0%) had ≥ 1 flare after TCZ initiation. PMR symptoms were reported in 7 of the 42 total flares (16.7%) before TCZ initiation and in 3 of the 14 total flares (21.4%) after TCZ initiation. TCZ was associated with a significant reduction in the annual flare rate in both patients with PMR symptoms (*p* = 0.003) and without PMR symptoms (*p* = 0.030) at diagnosis (Table 4). TCZ use was also associated with a significant reduction in the time to first flare among both patients with PMR at diagnosis (HR, 0.2; 95% CI, 0.1–0.5; *p* = 0.002) and without PMR at diagnosis (HR, 0.2; 95% CI, 0.1–0.9; *p* = 0.032; Supplemental Fig. S2).

**Table 4:** Disease flares in patients with and without PMR symptoms at diagnosis

	Patients with PMR symptoms at diagnosis (*n* = 32)		Patients without PMR symptoms at diagnosis (*n* = 28)	
	Before TCZ initiation	After TCZ initiation	Before TCZ initiation	After TCZ initiation
Follow-up time, median (IQR), years	0.7 (0.2–1.9)	0.6 (0.4–1.6)	0.6 (0.2–1.4)	0.5 (0.3–1.5)
Rate of flares per year*			1.6 (0.8–3.2)	
Rate (95% CI)	1.3 (0.8–2.1)	0.5 (0.3–1.1)		0.7 (0.3–1.7)
Rate ratio (95% CI)		0.4 (0.2–0.7)		0.4 (0.2–0.9)
*p* value		0.003		0.03
Patients with ≥ 1 flare, *n* (%)	24 (75.0)	11 (34.4)	19 (67.9)	7 (25.0)
With PMR symptoms^†^	21 (65.6)	8 (25.0)	4 (14.3)	2 (7.1)
Without PMR symptoms^†^	3 (9.4)	3 (9.4)	15 (53.6)	5 (17.9)
Total no. of flares	60	23	42	14
With PMR symptoms, *n* (%)^†^	50 (83.3)	18 (78.3)	7 (16.7)	3 (21.4)
Without PMR symptoms, *n* (%)^†^	10 (16.7)	5 (21.7)	35 (83.3)	11 (78.6)

*GCA* giant cell arteritis, *IQR* interquartile range, *PMR* polymyalgia rheumatica, *TCZ* tocilizumab

*Rates were estimated from a Poisson regression model with ongoing treatment (TCZ and glucocorticoid combinations), age, smoking history, and new or relapsing GCA as covariates, and random patient effect

^†^Symptoms were after GCA diagnosis. Percentage of flares is out of the total number of flares

### Outcomes in patients with visual manifestations at GCA diagnosis

Baseline characteristics for patients with and without visual manifestations at diagnosis are shown in Supplemental Table S2. Among the 22 patients with visual manifestations at diagnosis, 15 (68.2%) had a total of 29 flares before TCZ and 4 (18.2%) had a total of 9 flares after TCZ initiation (Table 5). Before TCZ, 9 patients (40.9%) in this subgroup had ≥ 1 flare with visual manifestations (blurred vision, *n* = 8; amaurosis fugax, *n* = 2; diplopia, *n* = 1; permanent vision loss/AION, *n* = 1). After TCZ initiation, 2 patients (9.1%) had ≥ 1 flare with visual manifestations (amaurosis fugax, *n* = 1; blurred vision, *n* = 2).

**Table 5:** Clinical outcomes in patients with and without visual manifestations at diagnosis

	Patients with visual manifestations at diagnosis (*n* = 22)		Patients without visual manifestations at diagnosis (*n* = 38)	
	Before TCZ initiation	After TCZ initiation	Before TCZ initiation	After TCZ initiation
Follow-up time, median (IQR), years	0.37 (0.20–0.92)	0.86 (0.28–1.16)	0.86 (0.18–1.84)	0.56 (0.37–1.73)
Rate of flares per year*				
Rate (95% CI)	1.2 (0.6, 2.5)	0.4 (0.1–1.2)	1.5 (0.9–2.4)	0.6 (0.3–1.2)
Rate ratio (95% CI)		0.3 (0.1–0.8)		0.4 (0.3–0.7)
*p* value		0.021		0.003
Patients with ≥ 1 flare, *n* (%)	15 (68.2)	4 (18.2)	28 (73.7)	14 (36.8)
Patients with ≥ 1 flare with visual manifestations, *n* (%)^†^	9 (40.9)	2 (9.1)	5 (13.2)	1 (2.6)
Permanent vision loss	1 (4.5)	0	1 (2.6)	0
AION	1 (4.5)	0	1 (2.6)	0
CRAO	0	0	0	0
Temporary vision impairment	9 (40.9)	2 (9.1)	5 (13.2)	1 (2.6)
Amaurosis fugax	2 (9.1)	1 (4.5)	2 (5.3)	0
Blurred vision	8 (36.4)	2 (9.1)	4 (10.5)	1 (2.6)
Diplopia	1 (4.5)	0	1 (2.6)	0
Total no. of flares	29	9	73	28
Total flares with visual manifestations, *n* (%)^†^	10 (34.4)	2 (22.2)	5 (6.8)	1 (3.6)
Permanent vision loss	1 (3.4)	0	1 (1.4)	0
AION	1 (3.4)	0	1 (1.4)	0
CRAO	0	0	0	0
Temporary vision impairment	10 (34.4)	2 (22.2)	5 (6.8)	1 (3.6)
Amaurosis fugax	2 (6.8)	1 (11.1)	2 (2.7)	0
Blurred vision	9 (31.0)	2 (22.2)	4 (5.5)	1 (3.6)
Diplopia	1 (3.4)	0	1 (1.4)	0

*AION* anterior ischemic optic neuropathy, *CRAO* central retinal artery occlusion, *GCA *giant cell arteritis, *IQR *interquartile range, *TCZ* tocilizumab

*Rates were estimated from a Poisson regression model with ongoing treatment (TCZ and glucocorticoid combinations), age, smoking history, and new or relapsing GCA as covariates, and random patient effect

^†^Symptoms were after GCA diagnosis. Percentage of flares is out of the total number of flares

Of the 38 patients without visual manifestations at diagnosis, 28 (73.7%) had a total of 73 flares before TCZ and 14 (36.8%) had a total of 28 flares after TCZ initiation (Table 5). Before TCZ, 5 patients (13.2%) in this subgroup had ≥ 1 flare with visual manifestations (blurred vision, *n* = 4; amaurosis fugax, *n* = 2; diplopia, *n* = 1; permanent vision loss/AION, *n* = 1). After TCZ initiation, 1 patient (2.6%) had ≥ 1 flare with visual manifestations (blurred vision, *n* = 1).

TCZ was associated with a significant reduction in the annual flare rate both in patients with visual manifestations at diagnosis (1.2 [95% CI 0. 6–2.5] before TCZ and 0.4 [95% CI 0.1–1.2] after TCZ; *p* = 0.021) and without visual manifestations at diagnosis (1.5 [95% CI 0.9–2.4] before TCZ and 0.6 [95% CI 0.3–1.2] after TCZ; *p* = 0.003). TCZ treatment was also associated with a significantly reduced time to first flare, in patients both with visual manifestations at diagnosis (HR 0.1 [95% CI 0.0–0.4]; *p* = 0.002) and without visual manifestations at diagnosis (HR 0.3 [95% CI 0.1–0.9]; *p* = 0.027; Supplemental Fig. S2).

## Discussion

This real-world data analysis confirms the effectiveness and safety of TCZ for GCA, complementing the results of recent randomized clinical trials [5, 9]. In this longitudinal routine-clinical-practice cohort, TCZ treatment was associated with a clinically meaningful reduction in the occurrence of disease flares. IL-6 receptor blockade therapy also led to glucocorticoid sparing, and no new safety signals were observed. In addition, our findings suggest that TCZ is an effective treatment for important subpopulations of patients with GCA, including those with visual manifestations and those with PMR symptoms at the time of disease onset. Moreover, our results demonstrate a reduction in the incidence of GCA-related visual manifestations occurring during disease flares after TCZ therapy initiation.

Assessment of the overall study population demonstrated that patients had significantly fewer flares and a significantly longer time to flare after TCZ initiation than before TCZ. In addition, the mean prednisone dose of this cohort was significantly lower by the end of follow-up than the dose at the time of TCZ initiation, and almost half of the patients were able to completely discontinue prednisone after initiating TCZ. The efficacy and glucocorticoid-sparing effects of TCZ in real-world patients with GCA shown in the present analysis are in agreement with the results of the GiACTA trial [5], as well as with what has been reported previously in other clinical trials [9] and case studies [10, 15–17]. The presence of PMR or visual symptoms at the time of GCA diagnosis did not influence the response to IL-6 blockade therapy. After TCZ initiation, patients had fewer flares and significantly longer times to flare regardless of the presence of PMR or visual symptoms at baseline.

Approximately one-third of patients with GCA report visual manifestations at disease onset [2, 3, 18]. These include diplopia, blurred vision, amaurosis fugax, and blindness, which occurs in 8 to 20% of patients, mostly due to AION [19–22]. Studies in patients treated with only glucocorticoids have reported that visual symptoms during disease relapse occur in approximately 5% of the patients and that permanent vision loss is rare [2, 3, 23]. A detailed evaluation of visual manifestations in patients with GCA receiving TCZ, however, has not been performed to date. Our results showed that GCA-related visual symptoms when patients were receiving only glucocorticoids (before TCZ was started) occurred during ≈ 15% of the flares. In contrast, once the patients were receiving IL-6 receptor blockade therapy, GCA-related visual manifestations were observed during only ≈ 8% of the flares. In addition, whereas 2 cases of AION were seen during a GCA flare before TCZ initiation, no cases of AION occurred after TCZ initiation. Of note, as described with glucocorticoid monotherapy, AION has been reported in a patient with GCA treated with TCZ 162 mg every 2 weeks [5]. In addition, transient visual symptoms concerning for ocular ischemia (e.g., amaurosis fugax and blurred vision) while receiving TCZ therapy were reported by 3 patients in our cohort. For this reason, clinicians should remain vigilant and monitor GCA patients for ophthalmologic complications even during treatment with TCZ.

Whether TCZ differentially controls PMR over other types of GCA clinical manifestations is currently unknown. To address this question, we analyzed the occurrence of flares with PMR symptoms before and after TCZ initiation. Whereas the proportion of patients with ≥ 1 disease flare and the total number of flares were reduced following TCZ introduction, PMR symptoms were seen in 55.9% and 56.8% of flares occurring before and after TCZ treatment, respectively. Therefore, although IL-6 inhibition is effective in treating GCA patients with PMR symptoms, it does not appear to specifically prevent the occurrence of PMR symptoms during disease relapse.

The proportion of patients with AEs before and after initiation of TCZ was similar (48.3% before and 53.3% after). In addition, an equal proportion of patients (10.0%) had SAEs prior to and during TCZ therapy. As expected, many of these AEs were attributed to glucocorticoids, which are known to cause toxicity in the great majority of patients with GCA [6–8, 24]. Similar to what was observed in the GiACTA trial [5], only a minority of patients (8.3%) required TCZ discontinuation due to an AE. The safety profile of TCZ in this analysis was consistent with the observed long-term safety profile across all approved TCZ indications [25].

This study had several notable strengths. First, the observational nature of the study provided real-world data on patient outcomes. An additional design-related strength is that each patient served as his or her own control because all patients were longitudinally evaluated before and after TCZ initiation. Finally, this study included, for the first time, analyses of response to real-world use of TCZ in subgroups of patients with visual manifestations or PMR symptoms at GCA diagnosis, as well as a detailed evaluation of visual manifestations and PMR symptoms during follow-up both before and after TCZ treatment. This study also had certain limitations. Because this was a retrospective assessment of real-world data, missing information and individual differences in the use of medications administered in routine clinical practice could have introduced some bias. Incomplete documentation of data related to individual prednisone tapering regimens made the calculation of cumulative prednisone dose, a key outcome measure in GCA, inaccurate and therefore not analyzable. This constraint was not surprising given the extended length of prednisone tapering used in the treatment of GCA and the frequent dose modification that may not only occur during clinical visits, but also in between visits. In addition, it is possible that some of the effects on disease relapse attributed to TCZ were instead related to the purported tendency of GCA to recur less often over time. This seems unlikely, however, in view of the striking difference in the number of flares and time to flare before and after TCZ use. Finally, given that the comparator population (i.e., pre-TCZ period) was enriched with flaring participants by virtue of the fact that the TCZ treatment decision was driven in most cases by disease flare, the reported reduction in flares could have been overestimated (violation of the self-controlled case series assumption). Despite these limitations, our present findings are consistent with, complement, and expand on the results from controlled clinical trials investigating the efficacy of TCZ in patients with GCA [5, 9].

## Conclusions

TCZ administered as SC or IV formulation improved clinical outcomes in patients with GCA, including those with visual manifestations or PMR symptoms at diagnosis, in a real-world setting as indicated by a reduction in the incidence of flares, increased time to flare, and robust reduction in the use of prednisone. TCZ reduced the occurrence of new GCA-related visual manifestations, and no new cases of AION or permanent vision loss occurred following TCZ initiation. The rates of AEs and SAEs, mostly attributed to glucocorticoids, were comparable before and after TCZ. These findings support the previously reported efficacy and safety of TCZ in patients with GCA and confirm that TCZ is an effective steroid-sparing treatment option for patients with GCA, including those with PMR symptoms and/or visual manifestations.

## Supplementary Information

**Additional file 1.**

## Data Availability

Qualified researchers may request access to individual patient-level data through the clinical study data request platform (http://www.vivli.org/). Further details on Roche’s criteria for eligible studies are available here: https://vivli.org/members/ourmembers/. For further details on Roche’s Global Policy on the Sharing of Clinical Information and how to request access to related clinical study documents, see here: https://www.roche.com/research_and_development/who_we_are_how_we_work/clinical_trials/our_commitment_to_data_sharing.htm.

## Abbreviations

AE: Adverse event
AION: Anterior ischemic optic neuropathy
CRAO: Central retinal artery occlusion
CRP: C-reactive protein
ESR: Erythrocyte sedimentation rate
GCA: Giant cell arteritis
HR: Hazard ratio
IL-6: Interleukin 6
IQR: Interquartile range
IV: Intravenous
PMR: Polymyalgia rheumatica
SAE: Serious adverse event
SC: Subcutaneous
TCZ: Tocilizumab
