# Supplementary material for: Clinical outcomes of patients with giant cell arteritis treated with tocilizumab in real-world clinical practice: decreased incidence of new visual manifestations
Source: Arthritis Res Ther. 2021 Jan 6;23:8. doi: 10.1186/s13075-020-02377-8 (PMC7789742; doi:10.1186/s13075-020-02377-8)
Supplement: Supplementary file 1 — Additional file 1. [file 13075_2020_2377_MOESM1_ESM.docx]

**Supplemental Material**

**Table S1** Patient baseline characteristics and treatments in patients with and without PMR symptoms at diagnosis

|  | Patients with PMR at diagnosis  (n = 32) | Patients without PMR at diagnosis  (n = 28) |
| --- | --- | --- |
| Age at diagnosis, mean (SD), years | 67.0 (9.27) | 71.8 (9.05) |
| White, n (%) | 27 (84.4) | 26 (92.9) |
| Female, n (%) | 23 (71.9) | 20 (71.4) |
| Current or previous smoking history, n (%) | 11 (34.4) | 10 (35.7) |
| New-onset disease, n (%) | 25 (78.1) | 23 (82.1) |
| Clinical manifestations at disease onset |  |  |
| Localized headache, n (%) | 28 (87.5) | 19 (67.9) |
| Scalp tenderness, n (%) | 15 (46.9) | 11 (39.3) |
| Jaw claudication, n (%) | 16 (50.0) | 15 (53.6) |
| PMR symptoms, n (%) | 32 (100.0) | 0 |
| Amaurosis fugax, n (%) | 5 (15.6) | 6 (21.4) |
| Transient blurry vision, n (%) | 9 (28.1) | 9 (32.1) |
| Diplopia, n (%) | 1 (3.1) | 1 (3.6) |
| Permanent vision loss, n (%) | 2 (6.3) | 6 (21.4) |
| Fever, n (%) | 7 (21.9) | 7 (25.0) |
| Weight loss, n (%) | 6 (18.8) | 14 (50.0) |
| ESR, mean (SD), mm/h | 67.9 (36.5) | 78.3 (29.4) |
| CRP, mean (SD), mg/L | 63.1 (59.7) | 92.4 (84.9) |
| Positive temporal artery biopsy, n (%)* | 13 (40.6) | 13 (46.4) |
| Large vessel vasculitis, n (%)* | 5 (15.6) | 7 (25.0) |

*CRP* C-reactive protein, *ESR* erythrocyte sedimentation rate, *PMR* polymyalgia rheumatica

*Of patients assessed.

**Table S2** Patient baseline characteristics and treatments in patients with visual manifestations at diagnosis

|  | Patients with visual impairments at diagnosis (n = 22) | Patients without visual impairments at diagnosis (n = 38) |
| --- | --- | --- |
| Age at diagnosis, mean (SD), years | 71.0 (11.0) | 68.2 (8.3) |
| White, n (%) | 18 (81.8) | 35 (92.1) |
| Female, n (%) | 16 (72.7) | 27 (71.1) |
| Current or previous smoking history, n (%) | 8 (36.4) | 13 (34.2) |
| New-onset disease, n (%) | 17 (77.3) | 31 (81.6) |
| Clinical manifestations at diagnosis |  |  |
| Localized headache, n (%) | 17 (77.3) | 30 (78.9) |
| Scalp tenderness, n (%) | 12 (54.5) | 14 (36.8) |
| Jaw claudication, n (%) | 15 (68.2) | 16 (42.1) |
| Visual manifestations at diagnosis |  |  |
| Amaurosis fugax, n (%) | 11 (50.0) | 0 |
| Transient blurry vision, n (%) | 18 (81.8) | 0 |
| Diplopia, n (%) | 2 (9.1) | 0 |
| Permanent vision loss, n (%) | 8 (36.4) | 0 |
| AION, n (%) | 7 (31.8) | 0 |
| Unilateral vision loss, n (%) | 4 (18.1) | 0 |
| Bilateral vision loss, n (%) | 3 (13.6) | 0 |
| CRAO, n (%) | 1 (4.5) | 0 |
| Unilateral vision loss, n (%) | 0 | 0 |
| Bilateral vision loss, n (%) | 1 (4.5) | 0 |
| Fever, n (%) | 5 (22.7) | 9 (23.7) |
| Weight loss, n (%) | 10 (45.5) | 10 (26.3) |
| PMR symptoms, n (%) | 10 (45.5) | 22 (57.9) |
| ESR, mean (SD), mm/h | 73.9 (26.3) | 71.8 (38.0) |
| CRP, mean (SD), mg/L | 78.2 (90.5) | 74.6 (59.2) |
| Positive temporal artery biopsy, n (%)* | 13 (59.1) | 13 (34.2) |
| Large vessel vasculitis, n (%)* | 3 (13.6) | 9 (23.7) |

*AION* anterior ischemic optic neuropathy, *CRAO* central retinal artery occlusion; *CRP* C-reactive protein, *ESR* erythrocyte sedimentation rate, *PMR* polymyalgia rheumatica

No significant differences in characteristics between those with and those without visual manifestations at baseline (*p* > 0.05 for all)

*Of patients assessed

**Fig. S1** Occurrence of individual disease flares while receiving glucocorticoids and/or TCZ


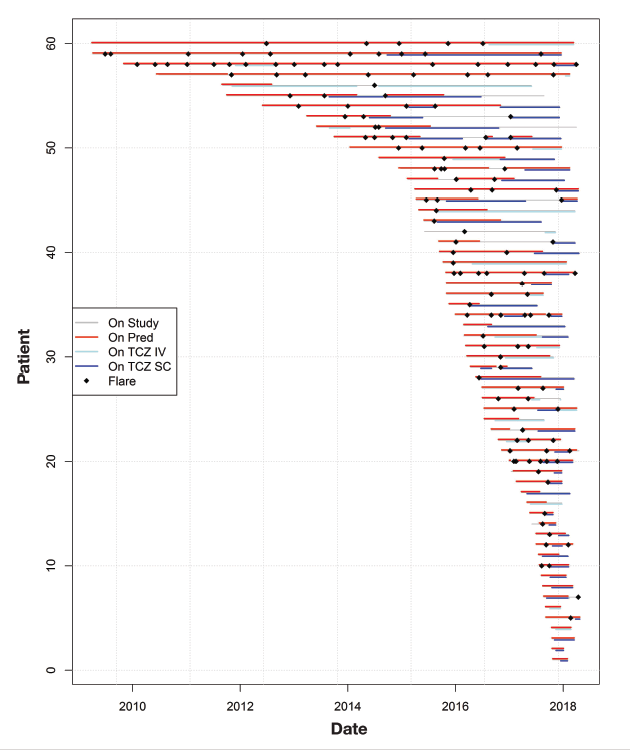


*IV* intravenous, *Pred* prednisone, *SC* subcutaneous, *TCZ* tocilizumab

**Fig. S2** Kaplan-Meier curves for time to first flare before and after TCZ initiation for patients (A) with PMR symptoms at GCA diagnosis and (B) without PMR symptoms at GCA diagnosis. Dotted lines represent 95% CIs

**
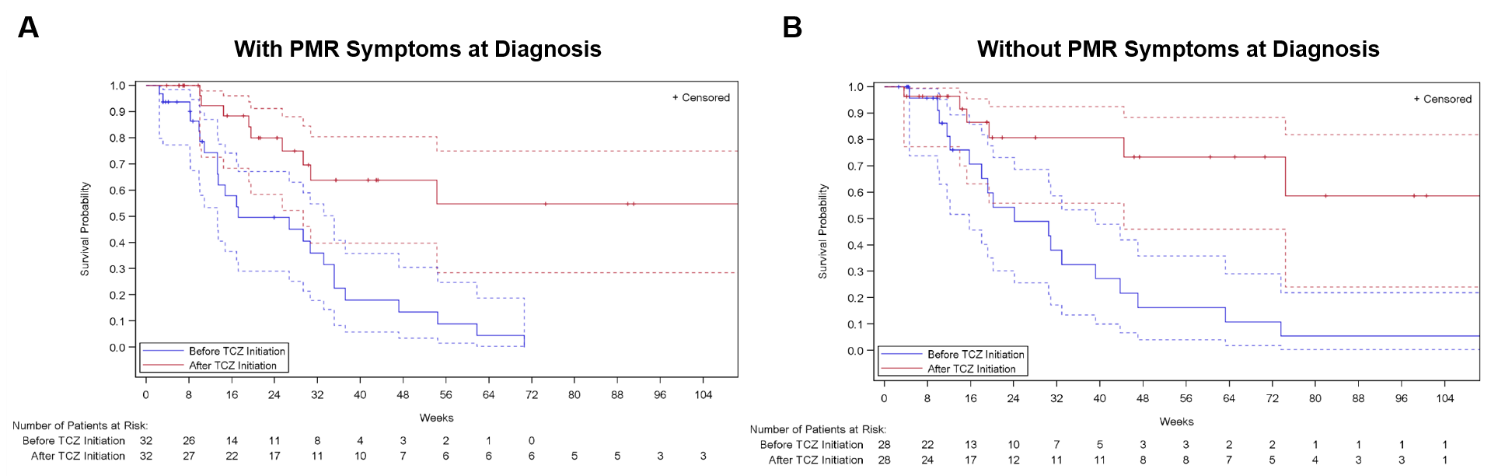
**

*GCA* giant cell arteritis, *PMR* polymyalgia rheumatica, *TCZ* tocilizumab

**Fig. S3** Kaplan-Meier curves for time to first flare for patients (A) with visual manifestations and (B) without visual manifestations at giant cell arteritis diagnosis. Dotted lines represent 95% CIs.

**
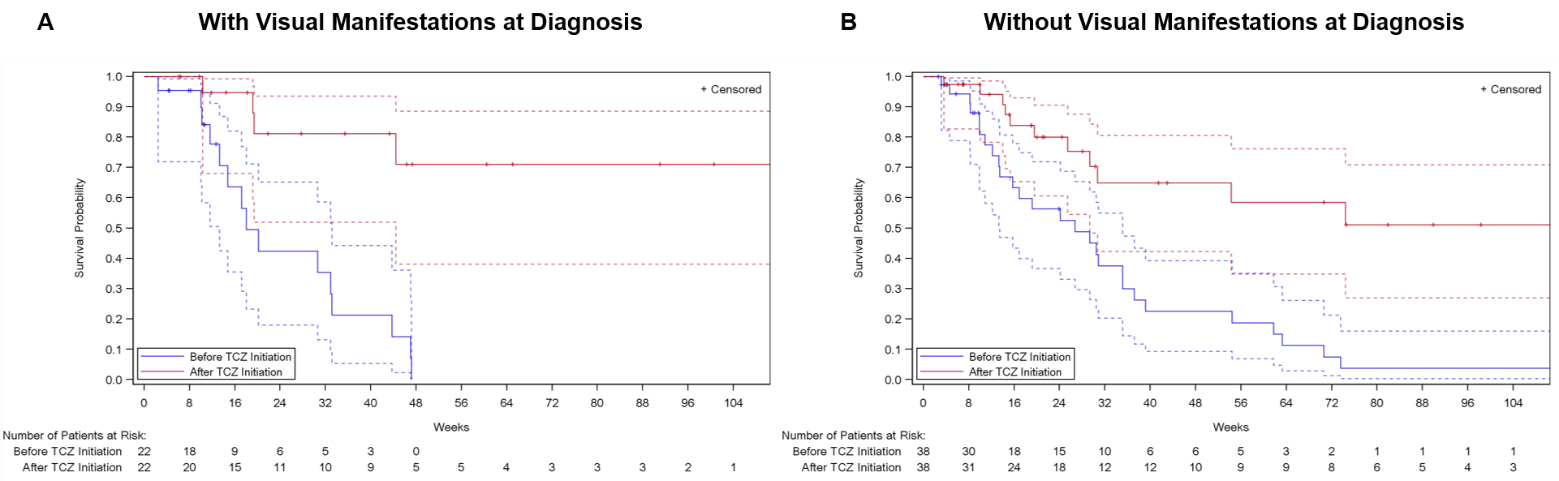
**

*TCZ* tocilizumab
